# Superior outcomes of pullout repairs for medial meniscus posterior root tears in partial tear compared to complete radial tear

**DOI:** 10.1186/s43019-023-00206-1

**Published:** 2024-02-08

**Authors:** Masanori Tamura, Takayuki Furumatsu, Yusuke Yokoyama, Naohiro Higashihara, Koki Kawada, Toshifumi Ozaki

**Affiliations:** Department of Orthopaedic Surgery, Okayama University Graduate School of Medicine, Dentistry, and Pharmaceutical Sciences, Okayama, Japan

**Keywords:** Knee injuries, Arthroscopy, Meniscus, Root tear

## Abstract

**Purpose:**

To reveal the outcomes of partial medial meniscus posterior root tears following transtibial pullout repair compared with the outcomes of complete radial meniscus posterior root tears.

**Materials and methods:**

We retrospectively evaluated 15 consecutive patients (male/female, 5/10; average age, 64.4 years) who underwent transtibial pullout repair for partial medial meniscus posterior root tears and compared their results with those of 86 consecutive patients who underwent the same surgery for complete medial meniscus posterior root tears. All patients underwent second-look arthroscopy on average 1 year postoperatively, and a semi-quantitative meniscal healing score (anteroposterior width, stability, and synovial coverage, total 10 points) was evaluated. Medial meniscus extrusion was evaluated preoperatively and at second-look arthroscopy.

**Results:**

Postoperative clinical scores were not significantly different in the short term. However, second-look arthroscopy revealed a significant difference in repaired meniscal stability (partial tear; 3.3 points, complete tear; 2.3 points, *p* < 0.001) and total meniscal healing scores (partial tear; 8.3 points, complete tear; 7.1 points, *p* < 0.001). Medial meniscus extrusion progression was significantly different (partial tear; 0.4 mm, complete tear; 1.0 mm, *p* < 0.001).

**Conclusion:**

Partial medial meniscus posterior root tears showed better meniscal healing and less medial meniscus extrusion progression following pullout repair than complete medial meniscus posterior root tears.

## Introduction

A medial meniscus (MM) posterior root tear (MMPRT) is a severe degenerative tear that mainly occurs in middle-aged people as a result of activities of daily living, such as descending stairs [1]. MMPRTs lead to pathological medial meniscus extrusion (MME) and result in the rapid progression of knee osteoarthritis. Compared to nonoperative treatments and meniscectomy, MMPRT repairs such as transtibial pullout repair can restore the meniscus's hoop tension, slow the progression of osteoarthritis, and prevent arthroplasty conversion [2, 3]. Although good clinical outcomes after complete MMPRT repair have been reported previously, MME progression occurs during the follow-up after pullout repair, especially in older patients with degenerative menisci [2, 4].

The grading of a meniscal root ligament lesion involves degeneration, partial tear, and complete tear [5]. A partial tear increases the local stress around the base of a crack [6] and could progress to a complete tear [7, 8]. In a partial MMPRT, the medial tibiofemoral joint stress increases by about 8.3% [6]. Moreover, in complete MMPRT, the peak contact pressure increases by about 25%, which is functionally almost compatible with total meniscectomy [9]. If physiological loading is applied for an extended period, the gap between the torn meniscus and root stump spreads, which could lead to further meniscus extrusion [10]. Some stable partial MMPRTs might heal naturally without a surgical procedure, and previously, conservative treatment was mainly performed in partial MMPRTs. However, a recent longitudinal study reported that MME progressed at approximately 0.46 mm per month after a painful popping episode in about half of the partial MMPRTs (25/48 knees) [11]. We thus assume that MMPRT repair in the early stage (partial MMPRT) is effective in some cases. However, little is known about the clinical outcomes of pullout repair for partial MMPRTs and the differences in clinical outcomes following pullout repair between partial and complete tears.

This study aimed to reveal the clinical, arthroscopic, and radiologic outcomes of partial MMPRTs following transtibial pullout repair and compare these results with those of complete radial MMPRTs. We hypothesized that the outcomes of pullout repair of partial MMPRTs are superior to those of complete radial MMPRTs because partial tears have some preserved remnants, which are beneficial in tissue regeneration and prevention of MME progression.

## Materials and methods

### Ethical approval and patient selection

**Fig. 1:**
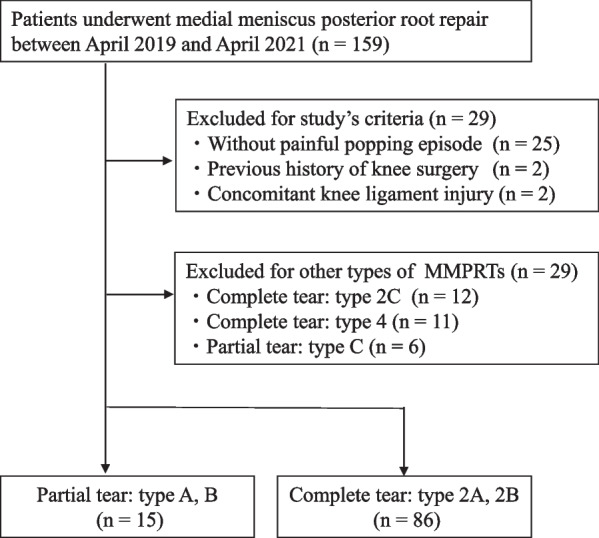
Flow chart showing the study protocol. MMPRT, medial meniscus posterior root tear

The study protocol was approved by the institutional review board of our hospital, and written informed consent was obtained from all participants. We retrospectively evaluated 15 consecutive patients with partial MMPRTs who underwent pullout repair between April 2019 and April 2021 (Fig. 1). Eighty-six consecutive patients with complete tears in the same period were evaluated as a control group. We compared the clinical outcomes between the groups. Partial tears were diagnosed based on the ocarina sign [12], and the absence of the following complete tear signs on magnetic resonance imaging (MRI): the cleft sign or giraffe neck sign [13] in the coronal view, the ghost sign in the sagittal view, and the radial tear sign in the axial view. Complete tears were diagnosed based on the presence of all the complete tear signs above. In both the partial and complete tear groups, transtibial pullout repair was indicated in patients with the following criteria: radiographic Kellgren–Lawrence grade 0–2 without subchondral insufficiency fractures, femorotibial angle ≤ 180°, mild cartilage lesions (Outerbridge grade I or II), and body mass index < 35 kg/m^2^. In the complete MMPRT group, early surgical repair within 3 months was recommended if patients met the surgical indications above [14]. However, in the partial MMPRT group, surgical repair was indicated if the patients had one of the following additional criteria: (1) continuous knee pain and dysfunction for more than 3 months (9 knees), (2) progression of MME during follow-up (3 knees, mean follow-up of 150 days), or (3) concomitant with major MME (> 3 mm) (7 knees).

During primary surgery, a complete radial tear was classified into three types based on the root tear position according to LaPrade’s classification: type 2A (0–3 mm from the root attachment center), type 2B (3–6 mm from the root attachment center), and type 2C (6–9 mm from the root attachment center) [15]. A partial tear was further classified into three arthroscopic morphologic types: type A, cleavage < 1/2 of the root width; type B, cleavage ≥ 1/2 of the root width; and type C, a complex horn tear expanding to the root, as reported by Furumatsu et al. [12]. This study excluded type 1C tears and type 2C tears because type 1A and 1B tears are arthroscopically similar to type 2A and 2B tears in terms of tear shape. The exclusion criteria of this study were as follows: no painful popping episodes, previous knee surgery on the injured side, and concomitant ligament injury. Details about sudden posteromedial painful popping episodes were obtained from the patients through interviews. All patients underwent arthroscopic second-look evaluation including the evaluation of the meniscal healing status 1 year postoperatively and MRI evaluation (Fig. 1).

### Surgical procedure and rehabilitation protocol

A well-experienced surgeon performed the diagnosis of MMPRT types and pullout repair for MMPRTs. Three different suture configurations were used in both groups according to the period of surgery: the two simple stitches using No. 2 polyethylene sutures with an additional posteromedial pullout technique (*n* = 61) between April 2019 and June 2020 [16], two cinch stitches (*n* = 19) using No. 2 polyethylene sutures between July 2020 and December 2021, and two cinch stitches with posterior anchoring (*n* = 21) between January 2021 and April 2021 [17]. In both groups, after limited debridement was applied at the tibial tunnel with a motorized shaver to obtain a path for a shuttle suture, a tibial tunnel was created, aiming at the root attachment center using aiming devices. The pullout sutures were fixed at the tibia using a bioabsorbable interference screw and tied under an anchor screw at a condition of 20–30° knee flexion angle with an initial tension of 10–30 N. Postoperatively, patients were asked to avoid weight bearing activities for 1–2 weeks, and eventually, partial weight bearing < 20 kg was allowed and gradually (+ 20 kg/week) increased until full weight bearing. Knee flexion exercise was initiated at 1–2 weeks postoperatively, and the flexion was gradually increased (+ 30°/week) to 120°. Patients were advised to avoid knee hyperflexion in weight bearing situations such as squatting, even after meniscal healing.

### MRI evaluations

**Fig. 2:**
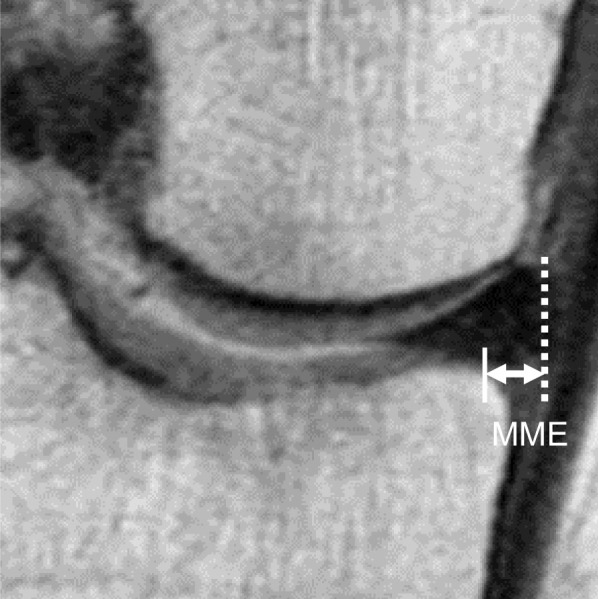
Measurement of medial meniscal extrusion (MME) (right knee). The extrusion was measured from the tibial edge (white line) to the outer edge of the meniscus (dashed white line) in the midcoronal plane

MRI evaluations were performed by two orthopedic doctors using Achieva 1.5T (Philips, Amsterdam, the Netherlands) with a knee coil. Sagittal (repetition time (TR)/echo time (TE), 742/18), coronal (TR/TE, 637/18), and axial (TR/TE, 499/18) images were obtained. A T2-weighted fast-field echo sequence was used with a 20° flip angle. The slice thickness was 3 mm, and the gap was 0.6 mm. The field of view was 16 cm (or 17 cm), with an imaging matrix size of 205 × 256 (or 200 × 368). MME was measured on a midcoronal plane that covered the largest area of the tibial spine, and osteophyte was excluded (Fig. 2) [18]. A second MRI was used to calculate the preoperative MME when patients underwent MRI examination twice before surgery. MME was evaluated preoperatively and during second-look arthroscopy. Progression of MME was defined as Δ MME, which was calculated by determining the difference between the pre- and postoperative MMEs. For further assessment, the suspension bridge sign (a characteristic MRI sign for good meniscal healing) [19] was evaluated.

### Computed tomography evaluation

Tunnel position was assessed using postoperative three-dimensional computed tomography (CT). The CT images were obtained with a Multislice CT system (Toshiba Medical Systems, Tochigi, Japan), and the three-dimensional images of the tibial condyles were reconstructed using a three-dimensional volume-rendering technique (AZE Virtual Place software; AZE Ltd., Tokyo, Japan). If the center of the bone tunnel was found to be within 5 mm of the virtual anatomic center of the MM posterior root attachment [20], it was considered an anatomic repair; otherwise, it was considered a non-anatomic repair.

### Clinical scores

Clinical scores were evaluated preoperatively and during second-look arthroscopy using the Lysholm knee score, Tegner activity score, International Knee Documentation Committee (IKDC) subjective knee evaluation form, Japanese Knee Injury and Osteoarthritis Outcome Score (KOOS), and pain score using a visual analog scale (VAS) ranging from 0 (no pain) to 100 mm (worst pain).

### Arthroscopic meniscal healing status and scores

All patients in both groups underwent second-look arthroscopy an average of 1 year after the primary surgery, and the semi-quantitative meniscal healing score of the MM posterior root was assessed [21]. This scoring system consists of three criteria: (i) the anteroposterior width of the bridging tissue between the MM posterior horn and root attachment, (ii) stability of the repaired MM posterior root, and (iii) synovial coverage of the sutured area.

**Fig. 3:**
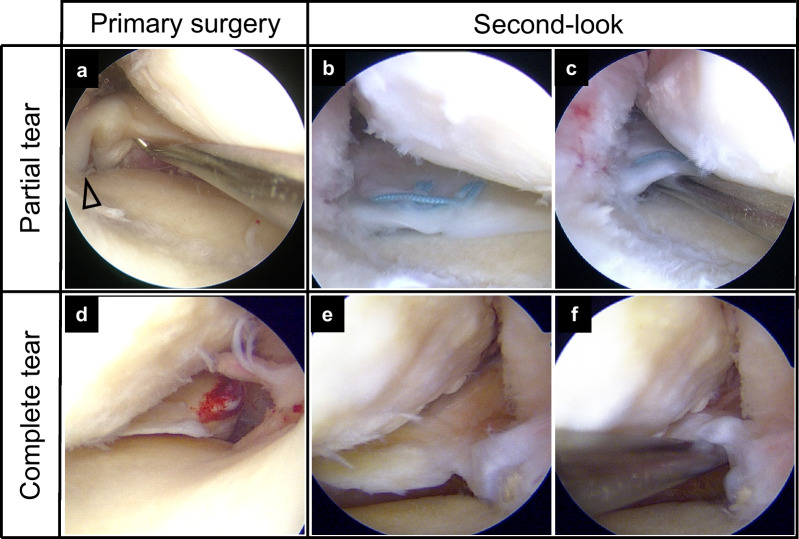
Arthroscopic findings at primary surgery and second-look arthroscopy. **A** Partial tear (type B) **B**, **C** Healed meniscus with good stability (4 points). **D** A complete tear (type 2A) **E**, **F** Healed meniscus with loose stability (2 points)

The width of the bridging tissue was evaluated as broad (> 5 mm, 4 points), narrow (2–5 mm, 2 points), or filamentous (< 2 mm, 0 points). The stability of the MM posterior root was evaluated as good (no lifting on probing at 20° knee flexion, 4 points), fair (lifting on probing during 20° knee flexion, but no lifting on probing at 60° knee flexion, 3 points), loose (lifting on probing at 60° knee flexion, but no anterior drawing at 20° knee flexion, 2 points), useless (presence of anterior drawing at 20° knee flexion, 1 point), and detached (totally unstable, 0 points). Synovial coverage was evaluated as good (2 points), fair (1 point), and poor (0 points). The total score ranged from 0 to 10 points (Fig. 3).

### Statistical analysis

Statistical analyses were performed using the EZR software (version 1.6–3) (Saitama Medical Center, Jichi Medical University, Saitama, Japan). Statistical significance was set at *p* < 0.05. The Mann–Whitney *U* test was used to compare the averages of continuous variables (such as age), and Fisher’s exact test was used to evaluate the proportions of categorical variables (such as sex). Wilcoxon’s signed-rank test was used to compare the pre- and postoperative clinical scores. Two observers independently evaluated MME. Each observer measured the values twice, at least 4 weeks apart. Intraobserver and interobserver correlations were assessed using intraclass correlation coefficients. The inter- and intraobserver reliabilities for the evaluations of MME were 0.88 and 0.85, respectively.

A post hoc power analysis to detect differences between groups in arthroscopic scores and Δ MME resulted in an actual power of 97.4% and 86.5%, respectively, with a significance level of 0.05.

## Results

**Fig. 4:**
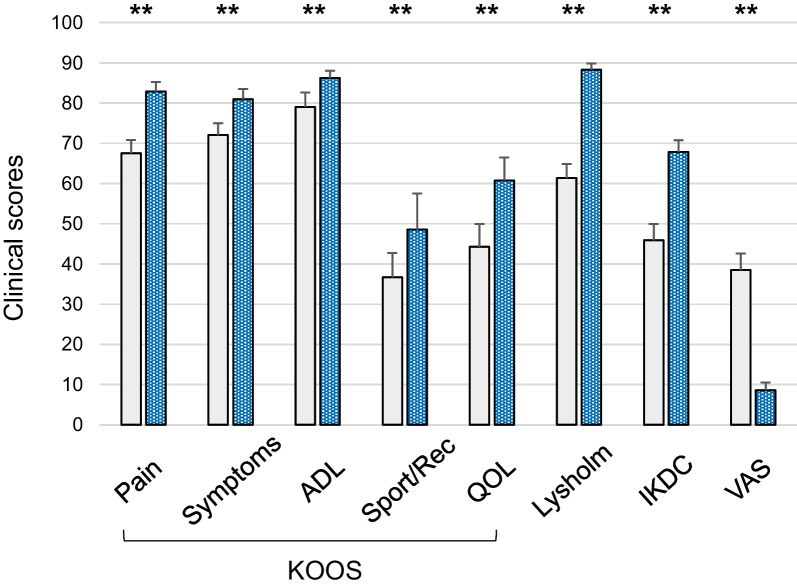
Pre- and postoperative clinical outcomes in the partial tear group. The shaded and blue dotted-line bars denote the preoperative and postoperative scores, respectively. **p < 0.05. *ADL* activities of daily living, *Sport/Rec* sport and recreation, *QOL* quality of life, *IKDC* International Knee Documentation Committee, *VAS* visual analog scale, *KOOS* Knee Injury and Osteoarthritis Outcome Score

All the clinical scores were significantly improved 1 year postoperatively compared to preoperative scores in the partial MMPRT group (Fig. 4). The patient demographic information is presented in Table 1. There were no significant differences between the two groups in terms of sex, patient age, height, body mass index, radiographic femorotibial angle, preoperative Kellgren–Lawrence grade, and surgical technique (Table 1). The duration from injury to surgery and from injury to preoperative MRI was significantly longer in the partial tear group than in the complete tear group (Table 1). Preoperatively, the IKDC and KOOS-QOL scores were significantly higher in the partial tear group than in the complete tear group (Table 2). Postoperatively, no significant difference in clinical scores was observed between the groups (Table 3). At second-look arthroscopy, the partial tear group had a better total meniscal healing score (partial tear; 8.3 ± 1.2 points vs. complete tear; 7.1 ± 1.1, *p* < 0.001) and stability subscale score (partial tear; 3.3 ± 0.9 points vs. complete tear; 2.3 ± 0.6, *p* < 0.001) than the complete tear group (Table 4). There was a significant difference in postoperative MME (partial tear; 3.2 mm vs. complete tear; 4.2 mm, *p* = 0.009) and progression of MME (partial tear; 0.4 mm vs. complete tear; 1.0 mm, *p* = 0.009) between the groups (Table 4). The suspension bridge sign was highly positive in the partial tear group compared to the complete tear group (Table 4).

**Table 1:** Patient demographics and clinical characteristics

	Partial tear (*n* = 15)	Complete tear (*n* = 86)	*p* value
Sex (male/female)	5/10	18/68	0.322
Age (years)	64.4 ± 7.4	66.4 ± 7.9	0.405
Height (m)	1.57 ± 0.1	1.54 ± 0.1	0.201
Weight (kg)	61.5 ± 11.1	62.1 ± 11.8	0.951
Body mass index (kg/m^2^)	25.0 ± 4.2	26.1 ± 4.7	0.597
Femorotibial angle (º)	178.0 ± 1.5	177.3 ± 1.6	0.698
Pre-operative K-L grade (0/1/2)	0/5/10	0/38/48	0.574
Surgical technique (TSS + PM /TCS/ TCS + PA)	10/2/3	51/17/18	0.925
Duration from injury to operation (day)	99.9 ± 80.1	54.2 ± 34.6	0.030*
from injury to pre-operative MRI (day)	73.2 ± 85.5	24.7 ± 30.9	0.032*
from pre-operative MRI to operation (day)	26.7 ± 15.2	29.5 ± 18.8	0.933
Partial tear classification (type A/B)	2/13		
Complete tear classification (type 2A/2B)		23/63	

Values are presented as mean ± standard deviation or number

*K-L* Kellgren–Lawrence, *TSS* two simple stitches, *PM* posteromedial pullout, *TCS* two cinch stitches, *PA* posterior anchoring, *MRI* magnetic resonance imaging *p < 0.05

**Table 2:** Comparison of preoperative clinical scores between groups

	Partial tear (*n* = 15)	Complete tear (*n* = 86)	*p* value
Lysholm knee score	61.4 ± 13.2	59.2 ± 11.6	0.070
Tegner activity score	1.9 ± 0.8	1.8 ± 0.9	0.070
IKDC score	45.9 ± 15.6	38.5 ± 15.3	0.040*
KOOS-Pain	67.6 ± 12.6	60.8 ± 16.9	0.321
-Symptoms	72.1 ± 11.4	64.4 ± 18.7	0.200
-ADL	79.1 ± 13.9	67.3 ± 15.7	0.010*
-Sport/Rec	36.7 ± 23.4	23.6 ± 23.6	0.018*
-QOL	44.3 ± 22.0	34.6 ± 18.9	0.101
Pain score (VAS)	38.5 ± 16.1	43.1 ± 27.0	0.809

Values are presented as the mean ± standard deviation

*IKDC* International Knee Documentation Committee, *KOOS* Knee Injury and Osteoarthritis Outcome Score, *ADL* activities of daily living, *Sport/Rec* sport and recreation; *QOL* quality of life, *VAS* visual analog score

*p < 0.05

**Table 3:** Comparison of postoperative clinical scores between groups

	Partial tear (*n* = 15)	Complete tear (*n* = 86)	*p* value
Lysholm knee score	88.4 ± 5.7	87.1 ± 6.3	0.634
Tegner activity score	3.0 ± 0.6	3.1 ± 0.5	0.631
IKDC score	67.9 ± 11.0	64.0 ± 15.2	0.613
KOOS-Pain	82.9 ± 9.1	86.0 ± 11.6	0.144
-Symptoms	81.0 ± 9.7	80.2 ± 15.1	0.915
-ADL	86.3 ± 6.7	86.8 ± 10.1	0.399
-Sport/Rec	48.6 ± 33.5	48.1 ± 30.5	0.955
-QOL	60.8 ± 21.3	62.7 ± 22.7	0.821
Pain score (VAS)	8.6 ± 7.1	11.1 ± 15.1	0.434

Values are presented as the mean ± standard deviation

*IKDC* International Knee Documentation Committee, *KOOS* Knee Injury and Osteoarthritis Outcome Score, *ADL* activities of daily living, *Sport/Rec* sport and recreation; *QOL* quality of life, *VAS* visual analog scale *p < 0.05

**Table 4:** Comparison of arthroscopic scores and MRI findings between groups

	Partial tear (*n* = 15)	Complete tear (*n* = 86)	*p* value
Arthroscopic scores			
Arthroscopic meniscal healing score (point) [range]	8.3 ± 1.2 [8, 9]	7.1 ± 1.1 [7, 8]	< 0.001*
Width of bridging tissues (point)	4.0 ± 0.0	3.9 ± 0.4	0.419
Stability (point)	3.3 ± 0.9	2.3 ± 0.6	< 0.001*
Synovial coverage (point)	1.0 ± 0.7	0.9 ± 0.5	0.252
MRI findings			
Pre-operative MME (mm)	3.0 ± 0.7 [2.5–3.6]	3.2 ± 0.9 [2.6–3.6]	0.598
3 M postoperative MME (mm) [range]	3.2 ± 0.8 [2.6–3.6]	3.5 ± 0.7 [3.1–4.0]	0.651
1 Y postoperative MME (mm) [range]	3.4 ± 1.0 [2.6–4.0]	4.2 ± 1.3 [3.7–4.9]	0.009*
ΔMME preoperative to 3 M (mm) [range]	0.2 ± 0.5 [0–0.5]	0.3 ± 0.5 [0.2–0.7]	0.213
ΔMME preoperative to 1 Y (mm) [range]	0.4 ± 0.6 [0.3–0.7]	1.0 ± 1.0 [0.5–1.6]	0.019*
Suspension bridge sign (positive/negative)	14/1	57/29	0.036*
Bone tunnel (anatomical/ non-anatomical)	14/1	76/10	1.000

Values are presented as mean ± standard deviation and first-third quartiles

*MRI* magnetic resonance imaging, *MME* medial meniscus extrusion, *3 M* 3-months, *1 Y* 1-year

*p < 0.05

## Discussion

The most important finding of this study was that pullout repair for partial MMPRTs effectively improved clinical outcomes and resulted in a better meniscal healing status, especially in stability, and lower MME progression compared to the results for complete tears. In contrast, no significant difference was observed in the postoperative clinical scores 1 year postoperatively.

To the best of our knowledge, this is the first study to demonstrate the effectiveness of pullout repair for partial MMPRT. However, the clear surgical candidate for partial MMPRT remains controversial. Meniscal root tears are a silent epidemic [22]. In symptomatic patients, posteromedial meniscus root ligament abnormalities, such as degeneration (14.3%), partial tear (11.7%), and complete tear (2.6%), are detected on MRI [5]. Arthroscopically, incomplete root tear was confirmed in about 16.4% [10] of the degenerative MMPRT cases in a previous study. The rate of surgical repair of partial tears was about 10% of all MMPRTs in our inclusion criteria. The percentage discrepancy might be because of the healing potential of partial MMPRT in its natural course and our strict selection for surgical repair. Nonoperative treatments such as non-steroidal anti-inflammatory drugs and physical therapy help relieve symptoms; however, osteoarthritis progresses in some patients [23]. Moreover, according to a previous study, in complete MMPRT, about 31% of the patients needed conversion to total knee arthroplasty after nonoperative treatment during a 5-year follow-up [24]. Although the partial tears of the root that progressed to complete tears were clinically unclear, the longer continuous knee disability in partial MMPRT in this study implies that the healing process was not sufficient in some partial MMPRT cases. A recent report revealed that major MME (> 3 mm) concomitant with partial MMPRT was a prognostic factor for MME progression in partial MMPRT. Furthermore, biomechanically, MME > 4 mm reduces the medial tibiofemoral compartment contact area [25]. Therefore, we considered that partial MMPRT with characteristics such as major MME between 3 and 4 mm, MME progression, and continuous pain might be a good surgical candidate.

**Fig. 5:**
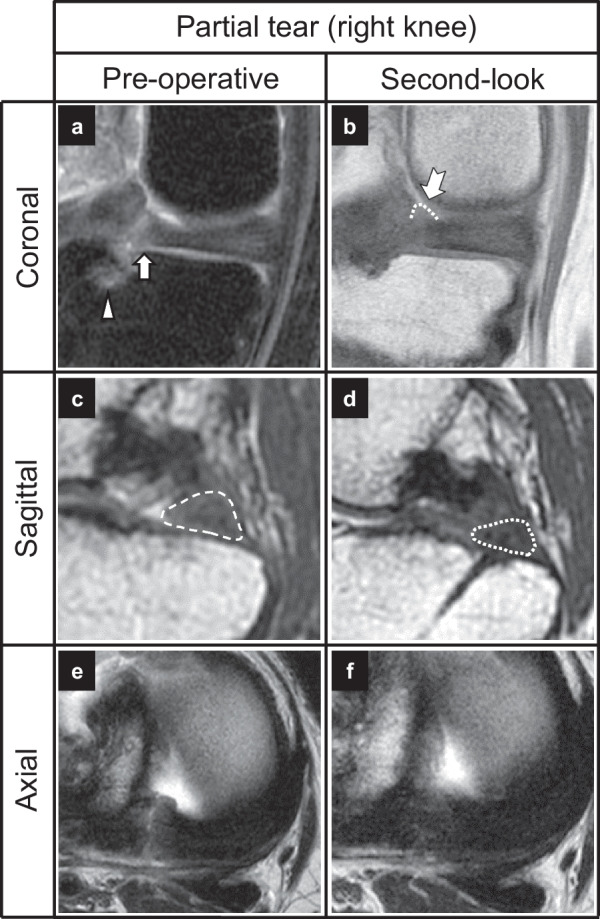
Pre- and postoperative MRI findings of a partial tear (same patient as in Fig. 3A–C). **A** Partial radial tear (arrow). **B** Suspension bridge sign (swallow-tail arrow). **C** Ocarina sign (dashed area). **D** Repaired meniscus (dashed line). **E** and **F** Axial images. *MRI* magnetic resonance imaging

**Fig. 6:**
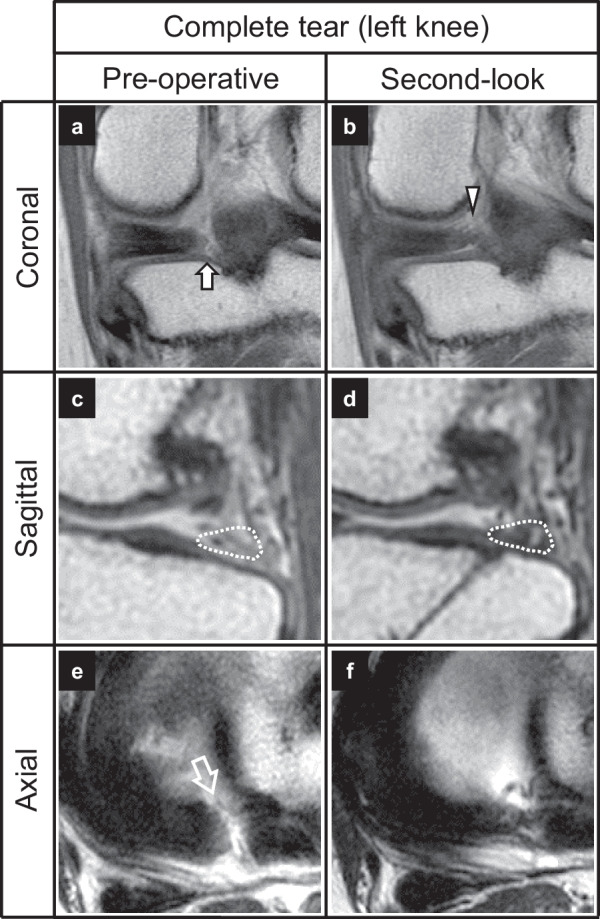
Pre- and postoperative MRI findings of a complete tear (same patient as in Fig. 3D–F). **A** Cleft sign (arrow). **B** No suspension bridge sign (arrowhead). **C** A ghost sign (dashed area). **D** Repaired root. **E** A radial tear sign (open arrow). **F** No radial tear sign. *MRI* magnetic resonance imaging

Early repair after symptom onset is critical in MMPRT management to prevent meniscus-induced osteoarthritis progression. Moon et al. reported that early surgical repair within 13 weeks from the onset of symptoms helped prevent the progression of MME [14]. Early detection of partial MMPRT is also important. In partial MMPRT, the ghost sign (or white meniscus sign) in the sagittal view and the radial tear sign in the axial view, found in complete tears, were not obvious in this study (Figs. 5, 6), and an ocarina sign was useful. However, other characteristic MRI signs of hyperintense bone marrow lesions, such as a spreading root sign [7] and posterior shiny corner lesion [26], are also reported. Familiarity with such signs would help diagnose partial MMPRTs and decision-making.

Although the clinical scores were significantly improved in both groups, MME worsened in both groups within the first postoperative year, with greater MME progression in the complete MMPRT group. Whether MME progressed preoperatively or postoperatively was difficult to determine in our study because an MRI was not performed in the immediate postoperative period. However, the accuracy of the bone tunnel was similar in both groups: 14/15 patients (93%) anatomically in the partial tear group and 76/86 patients (88%) in the complete tear group. Moreover, the 3-month postoperative MME was similar in both groups. Therefore, we assume that MME progressed over time, and the healing process might differ between groups. One possible reason for the difference was the grade of degeneration of the MM posterior root. In complete MMPRT, fibrocartilage metaplasia and calcification are more highly recognized at the tear site in a histological analysis [27], and a swollen, degenerated meniscus could be associated with a longer posterior shift during knee flexion by impingement between the femur and tibia [28, 29], which could lead to over-stress of the pullout sutures and could results in suture cut-out after pullout repair [30]. The lax meniscus healing in complete MMPRT might reveal that hoop tension was not completely restored. The optimal fixation technique and initial fixation condition, such as knee flexion angle and initial tension, should be further studied.

There was no significant difference in the synovial coverage subscale or width of bridging tissue subscale between the two groups at second-look arthroscopy. The pullout sutures were not always covered with synovial tissue, even in the partial tear group (Fig. 3), and this might have been caused by repetitive meniscus movement during the healing process. The similar width of bridging tissue score means that even in a complete MMPRT, meniscal healing can be obtained following pullout repair. However, greater stability of the repaired tissue and less progression of MME were achieved in the partial tear group than in the complete tear group (Fig. 3). We assume that the arthroscopic meniscus stability status could affect the hoop tension of the meniscus and how rapidly the arthritic changes progress, as MME has the potential to reflect cartilage degeneration and meniscus hoop tension [18]. One report revealed that the narrowing of the medial joint space width was smaller in the stable meniscal healing group than in the non-healing group following MMPRT repair [31]. In a future study, functional evaluation of meniscus hoop tension, such as meniscus extrusion in the weight-bearing knee or flexion positions on MRI or ultrasound, and arthritic changes over a longer period will be needed to confirm this hypothesis.

It is interesting that more lax healing in the complete tear group did not result in reduced clinical scores compared with the partial tear group. Previously, the semi-quantitative meniscal healing score system, which was used in this study, correlated with the KOOS QOL subscale following MMPRT pullout repair [21]. However, in our study, none of the clinical subscale scores showed a statistically significant difference between the partial and complete tear groups postoperatively. One possible reason was the low rate of poor meniscal healing (healing score ≤ 4 points) in this study. In the complete tear group, only one (1.2%, 1/86 knees in the complete tear group) patient had poor meniscal healing, although in a previous report, the rate was reported as 15% (3/10 knees) [21]. In addition, more moderate uniform healing occurred in the complete tear group; the first-third quartiles of the total healing score were 7–8 (Table 4), which was higher than that in a previous report [21]. In the present study, the surgical technique was gradually adjusted in an attempt to restrict abnormal meniscus movement when the knee was flexed [16, 17] and to improve the meniscal healing; however, second-look evaluation did not reveal any of the three suturing techniques to be superior to each other. Recently, several factors such as patient age [4], body mass index [32], time from injury to operation [14], appropriate bone tunnel position [20], and varus knee alignment [33] have been reported to be related to clinical outcomes following MMPRT repair. Appropriate surgical technique and patient selection may lead to avoid poor meniscal healing. Additionally, the weak relationship between the clinical scores and meniscal healing score and only one point difference in the meniscal healing score (partial tear group, 8.3 points vs. complete tear group, 7.1 points) may explain the lack of a significant difference in the clinical outcome subscale scores.

Although pullout repair for partial MMPRT could be a good therapeutic option, it also has problems. The lack of comparison with the natural history of partial MMPRT in this study may result in over-treatment of partial MMPRT. Additionally, the bone tunnel aperture and suture passing procedure could damage the remaining meniscus root fibers, so becoming accustomed to the surgical procedure is essential for repairing partial MMPRT. In the future, a prospective comparative study and longer follow-up period are needed to confirm the ideal patient selection and the advantages and disadvantages of surgical repair of partial MMPRT.

This study had some limitations. First, the sample size was small, and the retrospective nature of this study may have led to selection bias. Second, the follow-up period was short, and long-term follow-up is needed to assess differences in the clinical outcomes of MMPRT repair. Third, owing to the longer preoperative period in the partial MMPRT group, it was difficult to determine whether partial MMPRT represented incomplete healing after complete MMPRT or long-lasting partial MMPRT, which may affect the results. However, progression of MME (average 0.9 mm) after 150 days was found in three knees. In these three knees, the partial MMPRT remained without healing between the first and second preoperative MRIs. MME progressed even in patients with partial MMPRT over time. Those who have continuous knee pain with partial MMPRT could be good candidates for pullout repair. Fourth, differences in surgical technique were not found due to the small sample size in this study, which should be considered in further studies.

## Conclusions

Although the postoperative clinical scores were similar 1 year after surgery in both groups, transtibial pullout repair for partial MMPRTs demonstrated a better meniscal healing status with higher stability and less MME progression than transtibial pullout repair for complete radial MMPRTs.

## Data Availability

Not applicable.
